# Supplementary material for: Genetic basis and dual adaptive role of floral pigmentation in sunflowers
Source: eLife. 2022 Jan 18;11:e72072. doi: 10.7554/eLife.72072 (PMC8765750; doi:10.7554/eLife.72072)
Supplement: Supplementary file 1. [file elife-72072-supp1.zip › Supplementary file 1 HaMYB111_CDS_alignment.html]

BioJS viewer


 Launch in Jalview 


press "Run with JS"

  

Generated from
